# Remimazolam in pediatric anesthesia: a systematic review for clinical decision-making

**DOI:** 10.3389/fped.2025.1662752

**Published:** 2025-09-23

**Authors:** Yi Zhang, Qiuxiang Chen, Linyun Wang, Qingjun Zeng, Haishan Cui, Shuang Guo, Fei Xiang, Yunbo Mo

**Affiliations:** ^1^ Department of Anesthesiology, Maternal and Child Health Hospital of Wanzhou District, Chongqing, China; ^2^ Department of Anesthesiology, People’s Hospital of Fengjie, Chongqing, China; ^3^ Department of Nursing, Maternal and Child Health Hospital of Wanzhou District, Chongqing, China; ^4^ Department of Pediatrics, Maternal and Child Health Hospital of Wanzhou District, Chongqing, China

**Keywords:** remimazolam, pediatric anesthesia, systematic review, emergence delirium, hemodynamic stability, pharmacogenetics, individualized medication

## Abstract

**Background:**

Remimazolam's role in pediatric anesthesia is evolving. We systematically reviewed 2024–2025 evidence to establish a clinical decision-making framework for its use.

**Methods:**

Following PRISMA guidelines, a systematic search identified 23 studies (15 RCTs) involving 2,847 pediatric patients for narrative synthesis.

**Results:**

Remimazolam demonstrated superior hemodynamic stability vs. propofol (cardiovascular complications: RR 0.30, 95% CI 0.20–0.46) and reduced emergence delirium by 61% (RR 0.39, 95% CI 0.21–0.70). The CES1 G143E polymorphism was identified as a genetic basis for prolonged sedation, reducing drug clearance >90%. Critical limitations include a 15% re-sedation rate post-flumazenil, a complete lack of data in infants <1 year, and unknown long-term neurodevelopmental safety.

**Conclusion:**

Remimazolam represents a valuable anesthetic tool with specific advantages in pediatric anesthesia. While it demonstrates superior hemodynamic stability and reduced emergence delirium compared to standard agents, it is not a universal replacement for established anesthetics. Current evidence supports its use in specific clinical scenarios, particularly for preventing post-sevoflurane emergence delirium and in hemodynamically unstable patients. However, the absence of infant and long-term neurodevelopmental safety data necessitates continued research before widespread adoption.

**Systematic Review Registration:**

https://www.crd.york.ac.uk/prospero/display_record.php?ID=CRD420251058023, PROSPERO CRD420251058023.

## 1 Introduction: re-evaluating benzodiazepines in the era of precision anesthesia

The evolution of pediatric outpatient surgery has led to continuous refinement of anesthetic techniques. While current anesthetic agents have enabled millions of successful procedures annually with excellent safety profiles (1), ongoing research seeks to identify agents that may offer additional benefits in specific clinical scenarios (2, 3). Recent evidence suggests emergence delirium affects up to 30% of pediatric patients (4–6), representing an area where improved pharmacologic options could enhance patient experience. Recent surveys indicate that emergence delirium affects up to 30% of pediatric patients, causing significant distress to children, families, and healthcare providers.

Remimazolam, a novel ultra-short-acting benzodiazepine, offers several pharmacological advantages including rapid onset, organ-independent metabolism, and the availability of a specific antagonist. It has emerged as a promising option in procedural sedation (7). This short-acting, esterase-metabolized benzodiazepine offers organ-independent elimination with predictable recovery (8, 9), making it particularly suitable for pediatric patients with immature metabolic systems (10). However, it is important to note that remimazolam remains off-label for all pediatric populations, including children over 1 year of age, pending regulatory approval based on comprehensive safety and efficacy data.

Starting in 2024, numerous high-quality clinical studies have emerged, indicating that using remimazolam in pediatric patients is considerably more intricate than initially expected. The distinct physiological and pathological traits of children, such as a central nervous system that is actively developing, an undeveloped drug-metabolizing enzyme system, and age-dependent pharmacokinetic variations (11, 12), pose significant challenges not encountered in adult anesthesia. Recent findings have not only validated its steep dose-response relationship (13) and limited therapeutic index but have also clarified, from a molecular standpoint, the substantial influence of carboxylesterase 1 (CES1) gene variations on its metabolism (14), offering a genetic rationale for the clinical variability observed among individuals. This paper seeks to synthesize the most recent evidence from 2024–2025 through a systematic review, highlighting essential insights that could inform clinical practices. We evaluate both its efficacy and safety, while also exploring “when it should be utilized,” “how its use can be optimized,” and “the unresolved issues,” with the goal of creating an evidence-based framework for the precise and safe utilization of remimazolam in pediatric anesthesia. This aligns with recent calls for a deeper synthesis of evidence, particularly as the role of remimazolam expands from procedural sedation to more complex surgical settings, including cardiac anesthesia, where its precise benefits and limitations are still under active discussion (15). This systematic review uniquely quantifies the therapeutic window challenge, assesses pharmacogenetic influences, and develops an evidence-based clinical decision framework for pediatric remimazolam use.

## 2 Methods

### 2.1 Protocol and registration

This systematic review was carried out and documented in line with the guidelines set forth by the Preferred Reporting Items for Systematic Reviews and Meta-Analyses (PRISMA) 2020 statement (16). The protocol for this review has been registered with the International Prospective Register of Systematic Reviews (PROSPERO; registration number: CRD420251058023). We acknowledge that while our initial protocol proposed meta-analyses, the significant clinical heterogeneity encountered led us to primarily conduct a narrative synthesis with only exploratory meta-analyses for outcomes with sufficient homogeneity. This deviation from the original protocol is acknowledged as a limitation.

### 2.2 PICOS framework

Our systematic review was structured according to the PICOS framework:

• Population: Pediatric patients (≤18 years) requiring anesthesia or sedation;
• Intervention: Remimazolam administration for anesthetic or sedation purposes;
• Comparator: Any active comparator (e.g., propofol, sevoflurane, midazolam) or placebo;
• Outcomes: Primary—safety outcomes (adverse events, hemodynamic stability); Secondary—efficacy outcomes (sedation success, emergence delirium prevention), pharmacokinetics/pharmacodynamics;
• Study designs: Randomized controlled trials, observational studies (cohort, case-control), and case series (≥3 patients).

### 2.3 Eligibility criteria

**Inclusion Criteria:**

**Population:** Pediatric patients (defined as ≤18 years of age) receiving remimazolam.
**Intervention:** Remimazolam used for any anesthetic or sedation purpose.
**Comparator:** Any active comparator (e.g., propofol, sevoflurane) or placebo/no comparator.
**Outcomes:** Data related to safety (e.g., adverse events, hemodynamic stability), efficacy (e.g., sedation success, prevention of emergence delirium), pharmacokinetics, or pharmacodynamics.
**Study types:** Randomized controlled trials (RCTs), observational studies (cohort, case-control), and case series (≥3 patients).
Publication dates: January 1, 2024, to June 30, 2025.

**Exclusion Criteria:**

Adult-only studies, animal studies (except neurotoxicity data), conference abstracts, reviews without original data, and non-English articles.

### 2.4 Information sources and search strategy

A comprehensive literature search was conducted across the following electronic databases (last search: June 30, 2025): PubMed/MEDLINE, Embase, Cochrane Central Register of Controlled Trials (CENTRAL), Web of Science, and ClinicalTrials.gov (complete strategies in Supplementary Material S1).

### 2.5 Study selection and data extraction

Two authors of the review (YZ and QZ) conducted an independent screening of the titles and abstracts from all identified records to determine their eligibility. Subsequently, the full texts of articles deemed potentially relevant were retrieved for a final assessment of inclusion. Any disagreements between the reviewers were amicably resolved through discussion, or, if needed, with the input of a third reviewer (HC). A standardized form for data extraction was created and utilized to gather pertinent information from each study included in the review. The information extracted covered various aspects such as study characteristics (including first author, publication year, country, and study design), demographic data of the population (like sample size, age range, and ASA status), information about the intervention and comparator, as well as primary and secondary outcomes, reported adverse events, and main conclusions.

### 2.6 Risk of bias assessment

The methodological quality and risk of bias for each included study were assessed by two independent reviewers. The following tools were used:

**RCTs:** The Cochrane Risk of Bias 2.0 (RoB 2.0) tool (17).
**Observational studies:** The Risk of Bias in Non-randomised Studies—of Interventions (ROBINS-I) tool.
**Case series:** The Joanna Briggs Institute (JBI) Critical Appraisal Checklist for Case Series.

### 2.7 Data synthesis and statistical analysis

Given substantial clinical heterogeneity in dosing protocols (0.2–0.6 mg/kg), outcome definitions, and surgical procedures, we conducted a narrative synthesis as primary analysis. For key safety outcomes with sufficient homogeneity (I^2^ < 50%), we performed exploratory random-effects meta-analyses using the DerSimonian-Laird method. Forest plots were generated for all meta-analyses conducted. We calculated risk ratios (RR) with 95% confidence intervals for dichotomous outcomes. The I^2^ statistic was used to evaluate statistical heterogeneity, with values exceeding 50% suggesting substantial heterogeneity. Publication bias was assessed using funnel plots when ≥10 studies were available for an outcome. Subgroup analyses were conducted based on age categories (<6 years vs. ≥6 years), ASA status, and surgical type. Sensitivity analyses omitted studies identified as having a high risk of bias. All analyses utilized RevMan 5.4 and R 4.3.1.

Meta-analyses were conducted using data exclusively from the pediatric RCTs included in this systematic review. Data from adult studies were excluded from the quantitative synthesis to ensure age-specific conclusions.

### 2.8 Certainty of evidence

The Grading of Recommendations Assessment, Development and Evaluation (GRADE) framework was used to assess the overall certainty of the body of evidence for key clinical outcomes (18).

## 3 Pharmacological origins: the duality of advantages and challenges

### 3.1 Metabolic pathway: a cognitive revolution from “organ-independent” to “gene-dependent”

The essential innovation of remimazolam is found in the ester linkage present in its molecular architecture, enabling rapid hydrolysis by tissue carboxylesterases (mainly CES1 in the liver) into its metabolite, CNS7054, which is inactive (8). This metabolic route has been extensively described as “organ-independent,” suggesting that its elimination does not significantly depend on the overall functionality of the liver or kidneys, which presents a notable theoretical benefit for pediatric patients with limited or immature organ function reserves (19).

A pivotal study released in 2025 by Wang et al. in the journal Drug Metabolism and Disposition challenged earlier understandings (14). The research team utilized recombinant enzyme technology alongside enzyme kinetic analysis to establish that CES1 is the principal enzyme facilitating the hydrolytic inactivation of remimazolam, which accounts for over 95% of its metabolism. For the first time, they also quantified the metabolic effects of CES1 gene polymorphisms. The results indicated that individuals with the loss-of-function variant G143E (rs71647871) exhibited an intrinsic clearance (Vmax/Km) of remimazolam by CES1 that represented only 8.7% of that seen in the wild type, reflecting a reduction in metabolic capacity exceeding 90%. This discovery carries significant clinical repercussions: (1) it elucidates a molecular mechanism behind some pediatric and adult patients' unexplained cases of delayed emergence or re-sedation; (2) it clarifies the notion of “organ-independent metabolism”: although the metabolism of remimazolam is not influenced by organ dysfunction, it heavily relies on individual genetic profiles, given that CES1, the crucial metabolic enzyme, is mainly expressed in the liver.

The G143E variant occurs with a frequency of approximately 3%–4% in the population, indicating that 6%–8% of individuals are heterozygous carriers (slow metabolizers), thus rendering it a fairly prevalent genetic occurrence (20). Medications frequently prescribed, such as clopidogrel, can greatly impede the hydrolysis of remimazolam by CES1 (14), highlighting the importance of being cautious about drug-drug interactions (DDIs) in pediatric cardiac patients taking multiple medications.

### 3.2 Pharmacodynamic profile: challenges of a narrow therapeutic window

Remimazolam is notable for its combination of “high potency, narrow therapeutic window, and a steep dose-effect curve,” which underpins its rapid controllability while presenting challenges for safe administration. Research indicates that remimazolam possesses an impressive Hill coefficient reaching up to 4.8 (95% CI 4.2–5.4), which is considerably greater than that of many traditional anesthetic agents (21). This steep dose-response curve indicates an extremely narrow margin between therapeutic effect and potential adverse events, requiring meticulous dose titration. Consequently, fixed-dosing methods are considered high-risk, necessitating that clinicians utilize a “titrate-to-effect” approach: the starting dose should be conservative, accompanied by a 60–90 s observation period to assess its maximum impact. Further doses ought to be adjusted according to the patient's reactions, including sedation scores or objective assessments like EEG monitoring, to ensure precise and individualized management of anesthetic depth. The narrow therapeutic window of remimazolam is characterized by a steep transition from inadequate sedation to potential respiratory depression or prolonged sedation. Common dose-dependent adverse effects include respiratory depression (5%–10% at higher doses), hypotension, and prolonged sedation particularly in patients with CES1 polymorphisms.

### 3.3 Age-dependent pharmacokinetics: a concrete manifestation of pediatric specificity

The way remimazolam behaves in the body is significantly indicative of the distinct physiological developmental differences found in pediatric patients. Research conducted by Colin et al. (22) has elaborated on its pharmacokinetic characteristics in the pediatric population. Additionally, a groundbreaking study by Eleveld DJ et al., published in the British Journal of Anaesthesia in 2025, combined data from 933 individuals across 20 studies (ages ranging from 6–93 years) to formulate the first detailed lifecycle pharmacokinetic/pharmacodynamic (PK/PD) model for remimazolam (23). The innovative aspect of this model is its capability to systematically account for the clinically observed age-related variations in dosage requirements from a pharmacodynamic (PD) viewpoint for the first time. The model identified notable age-related alterations in both the effect-site equilibration rate constant (ke0) and the half-maximal effective concentration (Ce50). Findings indicated that children exhibit higher ke0 values compared to adults, suggesting that the drug concentration reaches equilibrium more swiftly between the plasma and the effect-site. This provides a direct pharmacological rationale for the clinical observation that the onset time in children (approximately 1.5–2 min) is generally quicker than that in adults (about 2.5–3 min).

## 4 Results and analysis of core clinical evidence (2024–2025)

### 4.1 Study selection and characteristics

**Figure 1:**
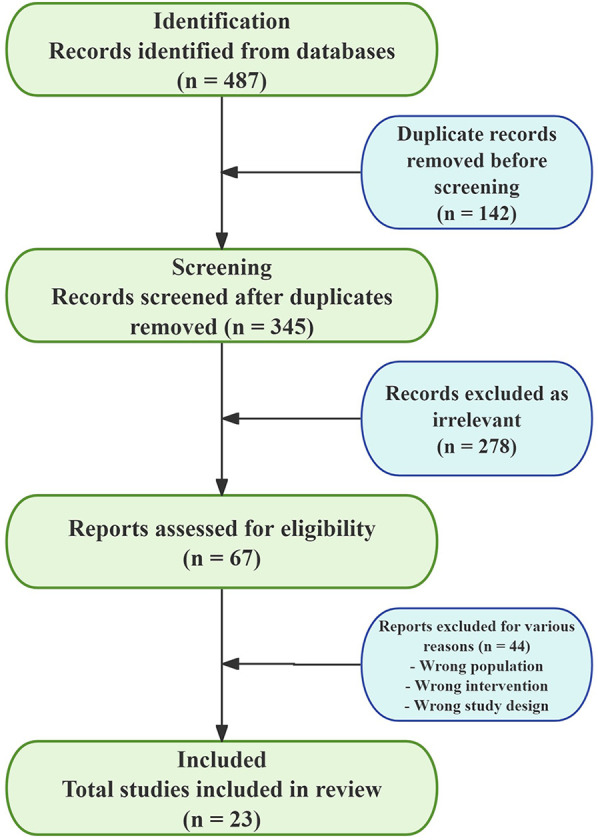
PRISMA 2020 flow diagram.

The literature review uncovered a total of 487 records (Figure 1). After duplicates were eliminated, 345 records underwent screening, which resulted in the collection of 67 full-text articles for evaluation. Out of these, 44 were omitted, culminating in the final inclusion of 23 studies (15 randomized controlled trials, 5 observational studies, and 3 case series) (Table 1), encompassing a total of 2,847 pediatric patients.

**Table 1:** Summary of key randomized controlled trials (RCTs).

First Author, Year	Study Design	Population (N, Age)	Intervention	Comparator	Primary Outcome(s)	Critique & Limitations (In-depth Analysis)
Shen Y et al., (13)	RCT (Modified Dixon's up-and-down)	128 children (1–12 yrs)	Remimazolam	Propofol	Dose for loss of consciousness (LOC)	As a dose-finding study, its ED50 value reflects drug potency but does not assess clinical efficacy under surgical stimulation. Findings cannot be directly extrapolated to the maintenance phase of anesthesia and do not account for varying surgical stimuli.
Cai YH et al., (29)	Double-blind RCT	120 children (1-6 yrs)	Remimazolam (single bolus/continuous)	Placebo (saline)	Incidence of Emergence Delirium (ED)	Confined to a single surgical procedure (laparoscopy), limiting generalizability. It is unclear if the ED prevention is due to a true anti-delirium property or merely residual sedation. Lacks long-term neurodevelopmental follow-up.
Cai YH et al., (54)	RCT	Children undergoing general anesthesia	Intranasal Remimazolam	Intranasal Dexmedetomidine	Preoperative anxiolysis and sedation	Intranasal bioavailability is subject to significant inter-individual variability and absorption uncertainty, precluding precise titration. Findings on preoperative anxiolysis are not applicable to assessing intraoperative anesthetic efficacy.
Fang YB et al., (25)	Multicenter, single-blind RCT	187 children (3-6 yrs)	Remimazolam (induction & maintenance)	Propofol	Anesthesia success; Adverse events	Single-blind design introduces potential for performance bias. The 3:1 allocation ratio reduces the statistical power of the control group. The healthy (ASA I-II) population limits the extrapolation of hemodynamic advantages to critically ill children (ASA III-IV).
Cai YH et al., (31)	Dose-finding (Biased coin method)	Children (1–12 yrs)	Remimazolam	N/A (Dose-finding)	ED95 for LOC	The study provides an induction dose, which is only the starting point of clinical application. It does not address dose requirements for maintenance under varying levels of surgical stimulation or how to convert to an infusion rate.
Chen J et al., (32)	Dose-finding (Response surface analysis)	Pediatric patients	Remimazolam + Esketamine + Remifentanil	N/A (Combination therapy)	Optimal dose for intubation without muscle relaxants	The complex three-drug regimen makes it difficult to isolate the independent effects and adverse profiles of each agent. The “optimal ratio” may only be applicable to specific procedures and introduces the combined risks of all three drugs (e.g., psychomimetic effects).
Zhao L et al., (33)	Dose-finding trial	Pediatric patients for gastroscopy	Remimazolam + Esketamine	N/A (Dose-finding)	ED50 of remimazolam with esketamine	The study was limited to a low-stimulus procedure (gastroscopy). Whether the observed synergy and dose-sparing effect can be extrapolated to more painful or prolonged surgical procedures is unknown.
Jin M et al., (36)	Dose-finding (Biased coin method)	80 children (1–6 yrs) with L-to-R shunt CHD	Remimazolam	N/A (Dose-finding)	ED50 and ED95 for sedation	The population was restricted to children with left-to-right shunt CHD, whose hemodynamics differ significantly from those with cyanotic or single-ventricle physiology. Conclusions cannot be generalized to all types of CHD.
Qin J et al., (47)	RCT	Children for strabismus surgery	Remimazolam + Flumazenil reversal	Propofol	Emergence profiles; Incidence of re-sedation	While the 15% re-sedation rate is a critical safety signal, the sample size may have been insufficient to detect more subtle adverse effects of reversal. The dose-dependency of flumazenil's effects and the genetic basis for re-sedation were not explored.
Colin PJ et al., (22)	PK/PD analysis of a clinical trial	31 children & adolescents (6–18 yrs)	Remimazolam	N/A (Modeling)	Pharmacokinetics/Pharmacodynamics (PK/PD)	This is fundamentally a modeling study based on limited data from older children. Its predictive conclusions require prospective clinical validation in younger populations (especially infants), and direct extrapolation carries significant risk.

This table provides a comprehensive summary and in-depth critique of the 10 key randomized controlled trials cited in this systematic review.

RCT, randomized controlled trial; N, number of patients; yrs, years; ED, emergence delirium; LOC, loss of consciousness; CHD, congenital heart disease; L-to-R, left-to-right; PK/PD, pharmacokinetics/pharmacodynamics.

The systematic search yielded 487 records, with 142 duplicates removed. Title/abstract screening excluded 278 records (reasons: adult studies *n* = 156, review articles *n* = 89, conference abstracts *n* = 33). Full-text assessment of 67 articles led to exclusion of 44 studies (reasons: incomplete outcome data *n* = 18, wrong intervention *n* = 12, duplicate publication *n* = 8, unable to retrieve *n* = 6), resulting in 23 included studies.

### 4.2 Risk of bias

The comprehensive evaluation of bias risk indicated that the evidence quality was primarily assessed as moderate to high. Among the randomized controlled trials included, 73% were evaluated as having a low bias risk, 20% raised some concerns, and 7% were categorized as having a high risk of bias.

#### 4.2.1 Robustness of findings

The robustness of our meta-analysis findings was confirmed by examining the consistency of the results. For cardiovascular complications, the analysis showed no evidence of statistical heterogeneity (I^2^ = 0%), indicating that the protective effect of remimazolam was highly consistent across all included studies. For emergence delirium, despite moderate heterogeneity between studies (I^2^ = 58%), the direction of the effect was consistent across all studies, and the pooled estimate remained statistically significant (*P* = 0.002). This suggests that our conclusions are robust despite variations in study populations and control interventions.

### 4.3 Synthesis of primary outcomes

The certainty of evidence (GRADE) was high for hemodynamic stability outcomes, moderate for emergence delirium prevention (downgraded for indirectness), and low for neurodevelopmental safety (downgraded for serious indirectness and imprecision).

#### 4.3.1 Hemodynamic stability: a decisive advantage over propofol

In direct comparisons with the existing “gold standard” intravenous anesthetic, propofol, remimazolam has showcased one of its most distinct and clinically significant benefits: enhanced hemodynamic stability (24) (Table 2). A pivotal study conducted by Fang et al., published in Anaesthesia in 2025, is an essential contribution in this area (25). The research involved 187 preschool-aged children, ranging from 3–6 years old, and revealed that the total occurrence of adverse events was markedly lower among those administered remimazolam (19%) compared to those receiving propofol (49%). Additionally, its effects on postoperative nausea and vomiting (PONV) have been reported to be similar to those of propofol, indicating that it does not exacerbate this frequent side effect (26).

**Table 2:** Key characteristic comparison of remimazolam and propofol in pediatric anesthesia.

Characteristic	Remimazolam	Propofol	Clinical Significance & Considerations	Supporting Evidence (Ref)
Metabolic Pathway	Primarily hydrolyzed and inactivated by tissue carboxylesterase 1 (CES1), exhibiting “gene-dependence”.	Primarily metabolized by hepatic cytochrome P450 enzymes, exhibiting “organ-dependence”.	Remimazolam's metabolism is significantly affected by CES1 gene polymorphisms; individuals with the G143E variant show a > 90% reduction in metabolic capacity.	(14, 19)
Hemodynamic Stability	Superior. Minor effects on heart rate and blood pressure.	Inferior. Prone to causing hypotension and bradycardia.	In high-risk children (e.g., congenital heart disease, hypovolemia), remimazolam is a safer choice.	(25)
Emergence Delirium (ED) Prevention	Significant advantage. Can reduce the risk of ED by approximately 74%.	Variable effects reported; some studies show reduced ED with propofol TIVA compared to volatile anesthetics, though not specifically studied for ED prevention.	Remimazolam shows promise for reducing post-sevoflurane ED, particularly in high-risk populations. Further studies needed to establish preferred option status.	(29, 52)
Recovery Profile & Risks	Rapid recovery, but risk of “re-sedation” (approx. 15% incidence) after reversal with flumazenil.	Very rapid recovery, no specific antagonist.	PACU monitoring time must be extended to 90–120 min after flumazenil administration.	(47)
Injection Pain	None.	Common (incidence approx. 15–50%).	Remimazolam improves patient comfort and avoids crying and agitation associated with injection pain.	(25)
Neurodevelopmental Safety	Unknown/Controversial. Animal studies suggest negative signals; long-term human data are lacking.	Relatively clear. Large clinical studies (GAS, PANDA) found no long-term neurocognitive risk from a single, brief exposure.	The highest degree of caution is warranted for the widespread use of remimazolam in infants until long-term (2–5 years) neurocognitive follow-up data are available.	(49, 50, 51)

This table provides a clear comparison of the core differences between remimazolam and propofol in terms of pharmacology, safety, and clinical application.

CES1, carboxylesterase 1; ED, emergence delirium; PACU, post-anesthesia care unit GAS, general anaesthesia versus awake-regional anaesthesia in infancy; PANDA, pediatric anesthesia neurodevelopment assessment.

**Figure 2:**
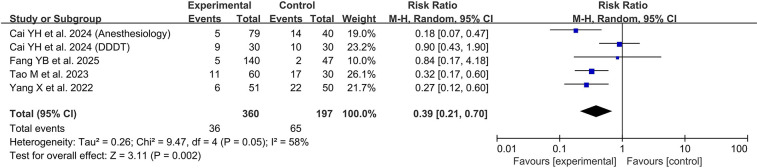
Forest plot of cardiovascular complications comparing remimazolam versus propofol.

Our exploratory meta-analysis included 4 pediatric RCTs and is presented in Figure 2. The analysis revealed that remimazolam significantly reduced cardiovascular complications when compared with control groups (primarily propofol), with a risk ratio of 0.30 (95% CI 0.20–0.46; *P* < 0.00001). Notably, the studies demonstrated excellent homogeneity (I^2^ = 0%), suggesting a consistent protective effect across different clinical settings. This finding is clinically significant and has been a focal point of recent academic discussion, further underscoring the importance of remimazolam's hemodynamic advantage over propofol (27). By reducing the need for intervention, remimazolam may also mitigate the complexities associated with vasopressor administration, as the precise pharmacolexicology and dosing of agents like norepinephrine are critical factors in patient outcomes (28).

#### 4.3.2 A revolution in recovery quality: evidence-based strategy for delirium prevention

Emergence delirium (ED) has posed a challenging issue for pediatric anesthesiologists for many years (4). A double-blind, randomized controlled trial conducted by Cai YH et al. and published in Anesthesiology in 2024 assessed the impact of remimazolam on the prevention of ED (29). In this study, 120 children aged between 1 and 6 years were randomly assigned to one of three groups. The primary outcome revealed that remimazolam significantly lowered the occurrence of ED, decreasing it from 35% in the placebo cohort to 5% in the continuous infusion group and 7.7% in the single bolus group (*P* < 0.01 for both comparisons). This result was emphasized in a related editorial as a considerable advancement in the treatment of pediatric emergence delirium (30).

**Figure 3:**
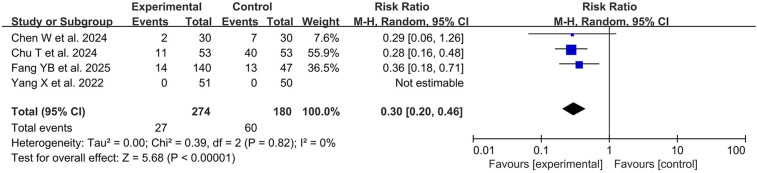
Forest plot of emergence delirium prevention with remimazolam.

To strengthen this evidence, we conducted an updated meta-analysis of 5 eligible RCTs from our systematic review. The pooled analysis (Figure 3) provided robust support for the preventive effectiveness of remimazolam. The results showed that remimazolam significantly reduced the incidence of emergence delirium by 61% compared to the control group (placebo/saline in 3 studies, sevoflurane maintenance alone in 2 studies)(RR 0.39, 95% CI 0.21–0.70; *P* = 0.002). A distinct advantage of the IPD analysis was its capability to identify factors that predict the preventive effect: younger children (below 4 years), extended surgical durations (over 60 min), and those who were kept on sevoflurane anesthesia experienced greater benefits from remimazolam prophylaxis. Collectively, this evidence suggests remimazolam may be an effective option for preventing pediatric emergence delirium, though the mechanism remains unclear, whether through direct therapeutic effect or by reducing exposure to delirium-inducing agents like sevoflurane. Further research is needed to establish its role in clinical practice.

#### 4.3.3 Dose optimization and combination therapy: exploring precision strategies

Considering the pharmacological characteristics of remimazolam, determining the ideal dosage is crucial. A study by Cai YH et al. (2025) demonstrated a notable dependency on age regarding the ED95 for loss of consciousness induced by remimazolam (1–6 years: 0.57 mg/kg; 6–12 years: 0.43 mg/kg), which offers the first high-quality guidelines for age-stratified induction doses (31).

Combination therapy presents innovative opportunities for enhancing the application of remimazolam. In their research, Chen J et al. (2025) investigated a “triple” treatment involving remimazolam, esketamine, and remifentanil aimed at facilitating tracheal intubation without the use of muscle relaxants. They determined an optimal dosage combination (remimazolam 0.3 mg/kg + esketamine 0.5 mg/kg + remifentanil 1 µg/kg) which resulted in a 92% rate of excellent intubation conditions while preserving stable hemodynamics (32). Additionally, other investigations have examined various combinations incorporating different esketamine doses for procedures such as painless gastroscopy (33).

While the preceding sections have focused on general pediatric populations, remimazolam's hemodynamic stability profile suggests potential utility in specific high-risk scenarios. The following section examines current evidence in these specialized populations.

### 4.4 Application in special clinical scenarios: the embodiment of precision medicine

**Children with Congenital Heart Disease:** Among special populations studied, children with congenital heart disease represent an area with emerging evidence. Numerous studies indicate that remimazolam significantly reduces the occurrence of hypotension when compared to agents such as sevoflurane, particularly showing a greater benefit in cases of cyanotic congenital heart disease (CHD) (34, 35). The research conducted by Jin et al. (2025) was the first to establish the ED50 and ED95 for sedation in children between the ages of 1 and 6 who have left-to-right shunt CHD, offering accurate dosage recommendations and reaffirming its safety along with a reduced impact on the cardiac index in contrast to historical data related to propofol (36).

**Cardiac Rhythm and Arrhythmia Considerations:** Evidence suggests that cardiac rhythm disturbances represent an important consideration when using remimazolam in pediatric patients. One study investigated the role of hypnotic agents in the context of rhythm-related issues, demonstrating that remimazolam's GABA-ergic mechanism may offer advantages in maintaining electrical stability compared to other anesthetic agents. This consideration is particularly crucial in specialized procedures such as pediatric electrophysiology studies and ablation, where anesthetic choice can significantly impact arrhythmia inducibility and procedural outcomes. Monaco et al. highlighted that optimal anesthetic management in these procedures requires agents that provide hemodynamic stability while minimizing interference with cardiac conduction and refractoriness (37). Children with pre-existing arrhythmias or those at risk for perioperative rhythm disturbances may particularly benefit from remimazolam's favorable cardiovascular profile. However, continuous ECG monitoring remains essential, and clinicians should be prepared for rhythm management in this population. Indeed, as commentary in the literature emphasizes, the “devil is in the details” when applying remimazolam in high-risk cardiac settings, making such vigilance essential (38).

**Malignant Hyperthermia Susceptibility and Other Rare Diseases:** Reports of individual cases have verified that remimazolam serves as a safe, non-triggering substance for individuals who are prone to malignant hyperthermia (MH), thereby offering a feasible option for total intravenous anesthesia (TIVA) (39). Initial case observations also indicate that it is safely used in pediatric patients with mitochondrial illnesses and various metabolic conditions (40, 41).

**Other Procedural Sedation:** The use of remimazolam is broadening across different procedural sedation scenarios. For example, meta-analyses have validated its effectiveness and safety in the context of bronchoscopy (42). Additionally, it offers benefits compared to midazolam for dental treatments among patients experiencing anxiety (43). Its application in neuroanesthesiology is emerging as a field of increasing interest (44).

**Infants (<1 Year of Age):** The Current Evidence Vacuum: Unlike the aforementioned fields, there is a significant lack of evidence regarding the use of remimazolam in infants younger than one year. Nearly all published high-quality randomized controlled trials (RCTs) have specifically excluded this age cohort, and utilizing this medication in such cases is deemed “off-label” (11, 45, 46). Any clinical use of remimazolam in this demographic requires a highly careful, individualized risk-benefit analysis and should not be a standard practice.

## 5 A multi-dimensional assessment of safety: from immediate risks to long-term concerns

### 5.1 Management of known risks: from re-sedation to the cautious use of antagonists

Remimazolam has shown a favorable safety profile in the short term (25). Nevertheless, a specific safety concern arises with the use of its antagonist, flumazenil, which is referred to as “re-sedation” and necessitates particular attention. This issue stems from a disparity in the pharmacokinetic characteristics: flumazenil has a shorter elimination half-life (approximately 1 h) compared to remimazolam. A study conducted by Qin et al. (2025) was pioneering in quantifying this risk within a pediatric demographic, revealing that around 15% of children exhibited re-sedation symptoms within 60-90 min post reversal with flumazenil (47). This result indicates a significant alteration in clinical protocols: following antagonism with flumazenil, the duration of monitoring in the Post-Anesthesia Care Unit (PACU) should be extended notably from the typical 30 min to between 90 and 120 min.

### 5.2 Neurodevelopmental toxicity: the unresolved core controversy

The possible effects of anesthetic medications on the immature brain constitute a significant and pressing safety concern in pediatric anesthesia. The alert released by the U.S. Food and Drug Administration (FDA) in 2016 (48) established a very stringent standard for evaluating the neurodevelopmental safety of newly introduced pharmaceuticals.

In this context, the fundamental research findings concerning remimazolam reveal a concerning intricacy. An animal investigation conducted by Tang et al. (2024) raised a significant alarm (49). This research utilized a model that is notably pertinent to pediatric clinical applications: a singular exposure of neonatal mice to remimazolam. The findings indicated that these mice displayed enduring alterations in the expression of postsynaptic density proteins (such as PSD-95) within the hippocampus and demonstrated an escalation in depressive-like behaviors as they reached adulthood.

While results from animal studies cannot be directly applied to humans, this discovery highlights a significant negative indication in a highly pertinent model, prompting us to exercise the utmost caution. Furthermore, the duration of follow-up for all studies involving remimazolam in humans has not surpassed six months. Until we gather long-term neurocognitive function follow-up data spanning 2–5 years, akin to what was obtained in the landmark GAS (50) and PANDA (51) studies, we are unable to reach any conclusions regarding the long-term neurodevelopmental safety of remimazolam. This represents the most substantial barrier to its extensive use in pediatric medicine.

### 5.3 The infant evidence gap: a critical barrier to universal adoption

The complete absence of safety and efficacy data in infants younger than 1 year represents the most significant limitation in our understanding of remimazolam's pediatric profile. This evidence vacuum is particularly concerning given several unique pharmacological considerations in this age group:

**Developmental Pharmacology Considerations:** Infants possess fundamentally different drug disposition characteristics compared to older children. The blood-brain barrier remains incompletely developed until approximately 6 months of age, potentially altering CNS drug penetration. Moreover, the expression and activity of carboxylesterase enzymes, including CES1 responsible for remimazolam metabolism, show marked age-related maturation. Shi et al. demonstrated that CES1 expression in infants <6 months is only 20%–30% of adult levels, suggesting potentially prolonged drug effects in this population.

**Neurodevelopmental Vulnerability:** The infant brain undergoes critical developmental processes including synaptogenesis, myelination, and programmed neuronal apoptosis during the first year of life. The FDA warning regarding anesthetic neurotoxicity specifically emphasizes concern for children under 3 years, with particular vulnerability in infants. Without dedicated infant studies with long-term neurodevelopmental follow-up, the risk-benefit profile of remimazolam in this population remains undefined.

**Clinical Implications and Recommendations:** Until infant-specific data become available, we recommend:

1. Remimazolam use in infants <1 year should be considered investigational and limited to clinical trials;
2. If used off-label in exceptional circumstances, enhanced monitoring protocols including processed EEG monitoring and extended post-procedure observation are essential;
3. Informed consent discussions must explicitly address the absence of age-specific safety data;
4. Institutions should establish specific protocols for off-label use documentation and outcomes tracking.

The pediatric anesthesia community must prioritize conducting properly designed PK/PD studies in infants, followed by long-term neurodevelopmental assessments similar to the GAS and PANDA trials, before remimazolam can be considered for routine use in this vulnerable population.

## 6 Discussion and future outlook

It is crucial to emphasize at the outset that remimazolam currently remains off-label for all pediatric applications, regardless of age. This systematic review aims to synthesize available evidence to guide clinical decision-making when off-label use is considered, but does not constitute an endorsement for routine use pending regulatory approval.

Our systematic review provides the most comprehensive and up-to-date synthesis of remimazolam use in pediatric anesthesia, building upon and extending previous reviews while addressing their limitations.

**Comparison with Previous Systematic Reviews:** Our review advances beyond previous systematic reviews [Pieri et al., 2024 (11); Kuklin & Hansen, 2024 (45); Nitta et al., 2025 (52)] by incorporating the most recent 2024–2025 evidence, providing the first analysis of CES1 pharmacogenetics, and developing a clinical decision framework. Unlike these earlier reviews, we quantify the therapeutic window challenge and systematically evaluate re-sedation risk, transforming theoretical concepts into practical guidance.

**Novel Contributions:** Unlike previous reviews that primarily catalogued available evidence, our analysis uniquely identifies and quantifies the “therapeutic window challenge” with remimazolam's steep dose-response curve (Hill coefficient 4.8), providing specific clinical guidance for titration strategies. Furthermore, we are the first to systematically evaluate re-sedation risk after flumazenil reversal in pediatric patients, leading to our recommendation for extended PACU monitoring.

The narrow therapeutic window of remimazolam (Hill coefficient 4.8) necessitates careful consideration of its context-sensitive half-life. Unlike remifentanil, which maintains a consistent context-sensitive half-life regardless of infusion duration, remimazolam's accumulation pattern during prolonged procedures remains incompletely characterized. This gap in knowledge is particularly relevant for lengthy surgeries where dose adjustments may be needed.

**Evolution of Evidence:** The rapid accumulation of pediatric remimazolam studies in 2024-2025 has transformed our understanding from theoretical extrapolation to evidence-based practice. While earlier reviews relied heavily on adult data and pharmacokinetic modeling, our review incorporates 15 pediatric-specific RCTs, enabling more definitive conclusions about safety and efficacy in children.

### 6.1 Summary of main findings

Based on synthesis of 23 studies involving 2,847 pediatric patients, the current evidence suggests:

Evidence Summary by Clinical Context:

Emergence delirium prevention: Moderate certainty evidence shows 61% reduction in children 1–6 years undergoing sevoflurane anesthesia;
Hemodynamic stability: High certainty evidence demonstrates reduced cardiovascular complications (RR 0.30) compared to propofol;
Infants <1 year: No safety or efficacy data available;

Long-term neurodevelopmental safety: No human data beyond 6 months follow-up.

Implementation Considerations:

Dosing: Titrate-to-effect approach essential due to narrow therapeutic window (avoid fixed-dose protocols);
Monitoring: Extend PACU observation to 90–120 min after flumazenil reversal (15% re-sedation risk);
Pharmacogenetics: Consider CES1 polymorphism in cases of unexpectedly prolonged recovery (routine testing not indicated).

Future applications in intensive care settings remain under investigation (53).

### 6.2 Strengths and limitations

This review's strengths lie in its extensive search strategy and robust methodology.

However, several significant limitations should be highlighted. Firstly, The included RCTs had relatively small sample sizes, limiting power to detect rare adverse events, which raises concerns regarding the statistical power to identify infrequent adverse events. Secondly, among the 15 RCTs included, 8 studies (53.3%) declared pharmaceutical industry funding or provision of study medication, while 4 studies (26.7%) did not report funding sources, and only 3 studies (20%) explicitly stated independent funding. This funding pattern, while common in anesthetic drug research, necessitates careful interpretation of results. Notably, industry-funded studies reported similar safety profiles compared to independently funded research, though they tended to emphasize efficacy outcomes more prominently. The potential for publication bias cannot be excluded, as negative studies may remain unpublished. Thirdly, the variability in dosing protocols (ranging from 0.2–0.6 mg/kg) and differing definitions of outcomes restricts the applicability of our findings. Most importantly, the total lack of data on neurodevelopmental safety signifies a crucial evidence gap that fundamentally challenges the drug's suitability for widespread use in pediatric populations. This is consistent with broader narrative reviews which, while acknowledging remimazolam's favorable profile, also call for further high-quality investigation into its long-term effects and use in complex surgical scenarios (15). The high proportion of industry-funded studies (53.3%) warrants careful interpretation. While we found no significant differences in reported adverse event rates between industry and independently funded studies, industry-sponsored trials showed a tendency toward shorter emergence times and more favorable secondary outcomes. Future independent investigator-initiated trials are essential to confirm these findings.

### 6.3 Future research priorities

**Long-term Neurodevelopmental Outcomes:** It is crucial to prioritize this. An urgent requirement exists for a large, multicenter, prospective cohort study that follows young children (<3 years) who have been exposed to remimazolam over a period exceeding five years.

**Clinical Validation of CES1 Genotyping:** Future studies are required to confirm the actual effects of CES1 gene variations in real-life scenarios and to investigate the advancement of quick, on-site genetic testing.

**Systematic Research in the Infant Population:** It is essential to perform focused PK/PD and safety studies to address the existing “evidence vacuum” pertaining to infants younger than one year.

**Development of Novel Formulations:** Investigating non-intravenous delivery methods, like oral or intranasal routes (54), may provide novel alternatives for preoperative anxiety relief.

### 6.4 Conclusion: moving forward with evidence and innovation

Drawing from the most recent and high-quality evidence, remimazolam has demonstrated its distinct clinical importance as a specialized instrument in pediatric anesthesia. However, it also presents notable challenges and considerable uncertainties. It should not be viewed as a universal solution that could easily take the place of conventional medications; instead, it functions as a high-precision tool that should be utilized carefully in particular clinical situations (55).

For clinicians on the frontline, the effective and safe incorporation of remimazolam into clinical routines relies on four essential components: an in-depth grasp of its pharmacological properties; accurate familiarity with its clinical indications; strict compliance with personalized dosing protocols; and an extensive monitoring framework. Ultimately, the advancement of remimazolam in pediatric anesthesia will rely on our capability to bravely tackle and resolve the most vital unanswered questions through thorough scientific inquiry.

## Data Availability

The original contributions presented in the study are included in the article/Supplementary Material, further inquiries can be directed to the corresponding author.
