# Supplementary material for: Remimazolam in pediatric anesthesia: a systematic review for clinical decision-making
Source: Front Pediatr. 2025 Sep 23;13:1662752. doi: 10.3389/fped.2025.1662752 (PMC12500429; doi:10.3389/fped.2025.1662752)
Supplement: Supplementary file 1 [file Datasheet1.pdf]

# Remimazolam in Pediatric Anesthesia: A Systematic Review for Clinical Decision-Making

## 1 Background & Challenge

- Up to 30% of pediatric patients experience emergence delirium
- Hemodynamic instability with current agents (propofol/sevoflurane)
- Need for safer anesthetic options in high-risk populations

## 2 Study Design

- Systematic review (2024-2025 literature)
- 23 studies analyzed (15 RCTs)
- 2,847 pediatric patients included
- PRISMA 2020 guidelines followed

## 3 Key Pharmacological Characteristics

- Short-acting benzodiazepine
- Metabolized by esterase (CES1)
- Organ-independent elimination
- Steep dose-response curve (Hill coefficient 4.8)

## Major Clinical Findings

**↓ 70%**

**Cardiovascular Complications**

Compared to propofol
(RR 0.30, 95% CI 0.20-0.46)

**↓ 61%**

**Emergence Delirium**

Compared to control group
(RR 0.39, 95% CI 0.21-0.70)

**15%**

**Re-sedation Risk**

After flumazenil reversal
(Requires extended monitoring)

**0**

**Infant Data**

Lack of safety/efficacy data
for infants <1 year

## ⚠️ Critical Safety Considerations

- **OFF-LABEL USE:** Remimazolam is not approved for any pediatric indication
- **Genetic Variability:** CES1 G143E polymorphism causes >90% reduction in metabolism
- **Unknown Long-term Safety:** No neurodevelopmental outcome data beyond 6 months
- **Evidence Gap:** Complete absence of data in infants <1 year

## Clinical Decision Framework

### Tier 1: Recommended

• ED prevention
• CHD patients
• MH susceptibility

### Tier 2: Consider with Caution

• Routine elective surgeries
• Known CES1 variants
(Requires careful titration)

### Tier 3: Avoid in Principle

• Infants <1 year
• Severe hepatic insufficiency
• Routine flumazenil use

## Future Research Priorities

- Long-term neurodevelopmental outcome studies (2-5 years follow-up)
- Dedicated infant (<1 year) PK/PD and safety studies
- Clinical validation of CES1 genotyping for personalized dosing
- Development of non-IV formulations for anxiolysis
